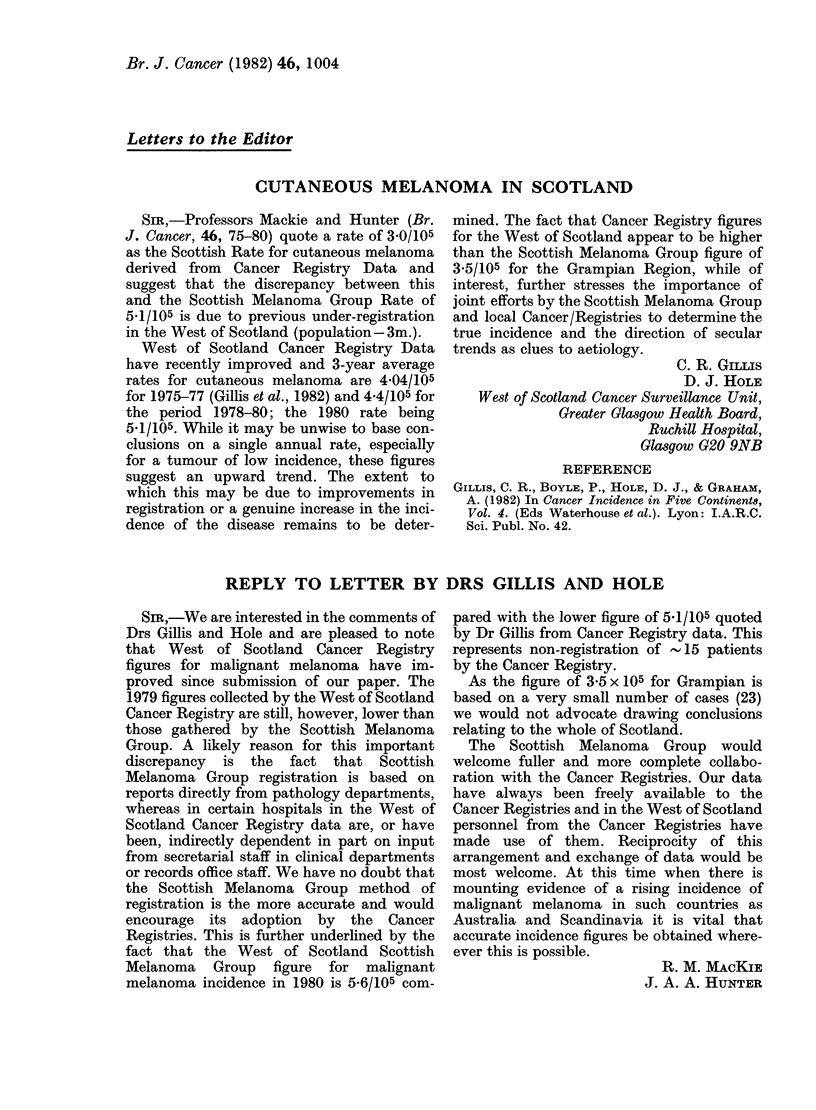# Cutaneous melanoma in Scotland.

**DOI:** 10.1038/bjc.1982.317

**Published:** 1982-12

**Authors:** C. R. Gillis, D. J. Hole


*Br. J. Cancer* (1982) **46**, 1004

### *Letters to the Editor*

### CUTANEOUS MELANOMA IN SCOTLAND

SIR,—Professors Mackie and Hunter (*Br. J. Cancer*, **46**, 75–80) quote a rate of $3{\cdot}0/10^5$ as the Scottish Rate for cutaneous melanoma derived from Cancer Registry Data and suggest that the discrepancy between this and the Scottish Melanoma Group Rate of $5{\cdot}1/10^5$ is due to previous under-registration in the West of Scotland (population – 3m.).

West of Scotland Cancer Registry Data have recently improved and 3-year average rates for cutaneous melanoma are $4{\cdot}04/10^5$ for 1975–77 (Gillis *et al.*, 1982) and $4{\cdot}4/10^5$ for the period 1978–80; the 1980 rate being $5{\cdot}1/10^5$. While it may be unwise to base conclusions on a single annual rate, especially for a tumour of low incidence, these figures suggest an upward trend. The extent to which this may be due to improvements in registration or a genuine increase in the incidence of the disease remains to be determined. The fact that Cancer Registry figures for the West of Scotland appear to be higher than the Scottish Melanoma Group figure of $3{\cdot}5/10^5$ for the Grampian Region, while of interest, further stresses the importance of joint efforts by the Scottish Melanoma Group and local Cancer/Registries to determine the true incidence and the direction of secular trends as clues to aetiology.

C. R. GILLIS
D. J. HOLE
*West of Scotland Cancer Surveillance Unit,*
*Greater Glasgow Health Board,*
*Ruchill Hospital,*
*Glasgow G20 9NB*

REFERENCE

GILLIS, C. R., BOYLE, P., HOLE, D. J., & GRAHAM, A. (1982) In *Cancer Incidence in Five Continents, Vol. 4.* (Eds Waterhouse *et al.*). Lyon: I.A.R.C. Sci. Publ. No. 42.